# Bidirectionally validated *in silico* and *in vitro* formation of specific depth zone-derived chondrocyte spheroids and clusters

**DOI:** 10.3389/fbioe.2024.1440434

**Published:** 2024-09-06

**Authors:** Eiichiro Takada, Hayato L. Mizuno, Yoshiki Takeoka, Shuichi Mizuno

**Affiliations:** Department of Orthopedic Surgery, Brigham and Women’s Hospital and Harvard Medical School, Boston, MA, United States

**Keywords:** *in silico*, cellular potts model, *in vitro*, spherical cell cluster model, longitudinal depth zone, articular cartilage

## Abstract

3D multicellular self-organized cluster models, e.g., organoids are promising tools for developing new therapeutic modalities including gene and cell therapies, pharmacological mechanistic and screening assays. Various applications of these models have been used extensively for decades, however, the mechanisms of cluster formation, maintenance, and degradation of these models are not even known over in-vitro-life-time. To explore such advantageous models mimicking native tissues or organs, it is necessary to understand aforementioned mechanisms. Herein, we intend to clarify the mechanisms of the formation of cell clusters. We previously demonstrated that primary chondrocytes isolated from distinct longitudinal depth zones in articular cartilage formed zone-specific spherical multicellular clusters *in vitro*. To elucidate the mechanisms of such cluster formation, we simulated it using the computational Cellular Potts Model with parameters were translated from gene expression levels and histological characteristics corresponding to interactions between cell and extracellular matrix. This simulation *in silico* was validated morphologically with cluster formation *in vitro* and *vice versa*. Since zone specific chondrocyte cluster models *in silico* showed similarity with corresponding *in vitro* model, the *in silico* has a potential to be used for prediction of the 3D multicellular *in vitro* models used for development, disease, and therapeutic models.

## 1 Introduction

Various *in vitro* 3D multicellular models including organoids have attracted great interest for recapitulating morphogenesis, metabolic turnover, and physiological functions of various organs in the human body (McCauley and Wells, 2017; Simian and Bissell, 2019). Cartilage organoids are one of them, demonstrated to be a powerful tool for investigating morphogenesis (Lamandé et al., 2023), pathogenesis (Dönges et al., 2024), and therapeutic modalities (Abraham et al., 2022). Development of such organoid models usually begins from clustering seeded cells without knowing the rationale of formation. This knowledge gap stems from a limited understanding of the dynamic formation, maintenance, and degradation of these organized multicellular models. To consistently explore the potential of such advantageous models, it is crucial to comprehend the mechanisms driving the formation of organized chondrocyte clusters.

We previously demonstrated that primary chondrocytes isolated from distinct longitudinal depth zones in articular cartilage form depth-zone-specific spherical cell clusters *in vitro* (Mizuno et al., 2016; Takada and Mizuno, 2018). The experimental methods employed in this study were similar to those used to form 3D multicellular models. Notably, unlike tumor cells, these chondrocytes produce abundant extracellular matrices (ECMs), which are essential for morphogenesis. Moreover, the quantity and composition of ECM varied significantly depending on the depth zone from which the chondrocytes were harvested. These findings indicate that depth zone–specific chondrocytes engage in cell-cell and cell-ECM interactions that generate the zone-specific configuration in spherical cell clusters.

In this study, to validate our hypothesis and uncover the factors contributing to the formation of unique clusters, we simulated the dynamic interaction between cells and their ECM during cluster formation in a computational (*in silico*) environment. We employed the Cellular Potts Model (CPM; Scianna and Preziosi, 2013; Voss-Bohme, 2012; Harjanto and Zaman, 2013; Li and Lowengrub, 2014), a tool for simulating fundamental cellular properties, and further defined interactive parameters including cell-cell, cell-medium, cell-ECM, and ECM-ECM affinities, as well as cell proliferation. These parameters were based on the gene expression of cellular and ECM molecules and the cytomorphological characteristics during chondrocyte spheroid/cluster formation *in vitro*. The *in silico* model is thus expected to closely mimic cellular behavior, making it a reliable tool for gaining deeper insights into the mechanisms of cluster formation. The simulation results obtained in this study strongly support the model’s effectiveness in replicating the formation of cell clusters derived from different cartilage depth zones, and provide valuable insights into the contribution of various ECM and their binding energies with neighboring cells and ECM to morphogenesis. The structure of our *in silico* model allows for the addition of diverse options, and with substantial parallel *in vitro* data sets to precisely program the robust behavior of cells and ECM in response to various environmental factors, it has the potential to simulate cellular behavior and ECM configuration without the need for case-by-case *in vitro* experiments. This study highlights the broad potential applications of an *in silico* model in recapitulating the interactive cellular and ECM behaviors underlying the formation and degradation of 3D multicellular models. The application of this *in silico* model will be invaluable for deepening our understanding of 3D cell-ECM interactions in tissue regeneration and pathogenesis, over extended timescales that surpass the observational limits of *in vitro* experiments.

## 2 Materials and methods

### 2.1 Cell isolation from specific depth zones in articular cartilage

**FIGURE 1:**
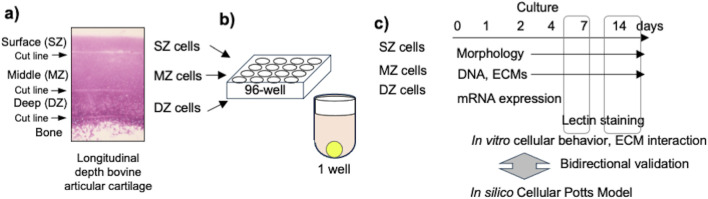
Experimental design. **(A)** Harvested bovine articular cartilage with cutline between adjacent depth zones in articular cartilage. **(B)** Seeded chondrocytes into round-bottom 96-well plate. **(C)** Incubation and sampling time course at 1, 2, 4, 7, 11, and 14 days after cell seeding.

To isolate articular chondrocytes from specific depth zones, forelimbs of 2–3-week-old calves were purchased from a local slaughterhouse (USDA certified) and delivered within 3 h of slaughter. We previously reported that this tissue already developed distinctive longitudinal depth cartilage tissue and isolated cells showed distinctive characteristics (Mizuno et al., 2016; Mizuno et al., 2016). Under aseptic conditions, cartilage pieces (<5 × 5 × 3–5 mm) were harvested from the distal condyle of the forelimb (Figure 1A). Zone-specific cartilage was isolated by slicing the SZ (100–200 µm thick) from the surface of the cartilage and DZ (200–300 µm thick) from the subchondral bone with a scalpel (#15, BD, Franklin Lakes, NJ) under a dissection microscope (Nikon, Nikon Instruments, Melville, NY). The remainder was defined as MZ and trimmed from each surface with enough margin (approximately >100 µm). Collagenase CLS 1 (Worthington, Lakewood, NJ) was dissolved at 0.15% in Ham’s F-12 medium (Invitrogen, Carlsbad, CA) and sterilized with a 0.45 µm filter (Nalgene™, Fisher Scientific, Hampton, NH). The slices were minced and gently digested in the collagenase solution with 100 units/mL penicillin and 100 μg/mL streptomycin (Invitrogen) at 37°C for 12 h on a rotator shaker (15 rpm). The dispersed bovine articular chondrocytes (bACs) isolated from each zone were strained with a 70-µm-cell strainer (Falcon^®^) to remove debris. The cells were rinsed twice with Dulbecco’s phosphate-buffered saline (D-PBS, Invitrogen) and then suspended in Dulbecco’s modified Eagle medium/Ham’s F-12 (1:1) medium (DMEM/F12) with 10% fetal bovine serum (Life Technologies), 100 units/mL penicillin, and 100 μg/mL streptomycin (Invitrogen). Harvesting a specific zone tissue may have a risk of including adjacent tissue. We defined the population as single cell source because these populations interact in a native tissue.

### 2.2 Cell seeding and spheroidal cluster formation

**FIGURE 2:**
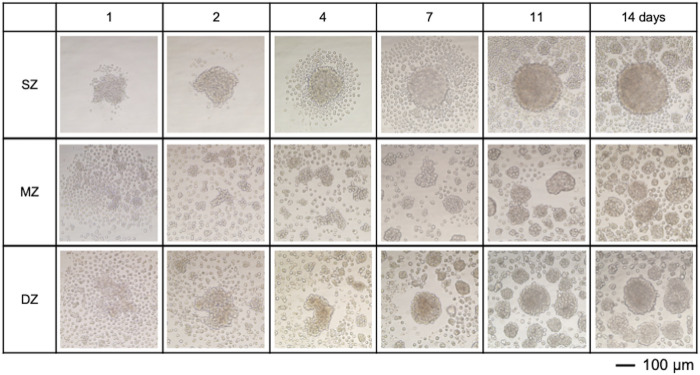
Spheroids formed by chondrocytes isolated from longitudinal depth zones in bovine articular cartilage at 1, 2, 4, 7, 11, and 14 days after cell seeding. Surface-zone-derived spheroids/cell clusters; SZ, Middle-zone-derived spheroids/cell clusters; MZ, Deep-zone-derived spheroids/clusters; DZ. The bar indicates 100 µm.

One thousand chondrocytes each were isolated from SZ, MZ, and DZ and seeded in 150 µL of DMEM/F12 medium to a round-bottom/low cell-adherent well of a 96-well plate (Prime-Surface™ S-BIO, Hudson, NH) and incubated at 5% CO_2_ in air, at 37°C for 2 weeks (Figure 1B, C). The cells were incubated without changing the medium over the course of the experiments to avoid disturbance. The culture medium may create hashed conditions. We designed this approach for *in silico* simulation based on preliminary experiments and our prior work. During the 14-day culture, less than 10% of the culture medium evaporated in a 95% humidity incubator. The outermost rows and columns of the 96-well plate were not used, since medium evaporation from these wells was notably large. Images of 16 randomly chosen wells out of 60 were acquired at 12, 24, and 48 h and 1, 4, 7, and 14 days using an inverted microscope (TMD, Nikon Instrument; D80, Nikon) and a camera (Figure 2). Cell viability in the spheroids was evaluated using a cell viability assay kit (Life Technologies, Carlsbad, CA). Briefly, the spheroids were incubated with 4 μM calcein AM and 4 μM ethidium homodimer dissolved in D-PBS at room temperature for 30 min. The spheroidal clusters were then fixed with 2% paraformaldehyde/10 mM phosphate-buffered saline (pH 7.4) and observed at excitation 459–490 nm and emission at >515 nm, and at excitation 510–560 nm and emission at >590 nm, respectively using an inverted microscope.

### 2.3 Production of sulfated glycosaminoglycan and amount of DNA

At 1, 4, 7, and 14 days, suspended non-adherent cells and cell clusters were collected from each well and transferred to 1.5-mL microtubes, then centrifuged at 0.2 rtf (5415D, Eppendorf, Enfield, CT) for 5 min. The supernatant was slowly removed using a fine pipette tip under a dissection microscope (SMZ-1, Nikon) and transferred to another 1.5-mL microtube. The pellets and medium were frozen at −80°C until DNA and sulfated glycosaminoglycan (S-GAG) assays were conducted. For DNA assay, the pellets were frozen and thawed twice to disturb the cell membrane, then dissolved in 100-µL Tris-buffer (50 mM Tris-HCl, 100 mM NaCl, and 0.1 mM ethylenediaminetetraacetic acid, pH 7.4) and mixed with 100-µL Hoechst 33258 at 1.0 μg/mL dissolved in the Tris-buffer. After incubation avoiding light at room temperature for 15 min, fluorescence intensity was measured using a fluorometer (TBS-380, Turner Biosystems, Sunnyvale, CA). For the S-GAG assay, 30 µL of the culture medium was mixed with 70 µL of dimethyl methylene blue (DMMB) solution (5 mg in formic acid, pH 4.0) in a 96-well micro-titer plate, and the optical density of the sample was immediately measured at 540 nm using a microplate reader (iMark, Bio-Rad, Hercules, CA).

### 2.4 Identification of chondroitin sulfate and hyaluronan in cell clusters with fluorescent lectins

At 14 days, the culture medium was gently removed from the upper phase of the medium in each well, and 200 µL of 2% paraformaldehyde/10 mM D-PBS was added into each well to fix the cells. The fixative was removed in the same manner as described above, then 200 µL of fresh fixative was added into each well to avoid fixative dilution. The cells and clusters were transferred to a 200-µL clear plastic sample tube and rinsed with D-PBS using a microcentrifuge at 0.2 rtf for 15 s. After gently aspirating supernatant, the cells/clusters were incubated in 200 µL of 40 μg/mL Wisteria floribunda lectin (WFL)-Fluorescein (Vector Laboratory, Newark, CA) to bind specifically to *N*-acetylgalactosamine and illuminate CS composed of a chain of alternating sugars (*N*-acetylegalactosamine and glucuronic acid). Similarly, the cells and clusters 200 µL of 50 μg/mL wheat germ agglutinin lectin (WGA)-Alexa Fluor-594 (Invitrogen, Waltham, MA) for 30 min to specifically bind to *N*-acetylneuraminic acid and *N*-acetlylglucosamine, and to illuminate hyaluronan (HY, *N*-acetylglucosamine and glucuronic acid), then counter-stained with 2 μg/mL diamidino-2-phenylindole (DAPI, Invitrogen) to illuminate the nuclei. The cells were then rinsed with D-PBS. The cells/clusters were rehydrated with graded 30, 50, 70, 90, and 100% cold ethanol. Before changing the ethanol, the tubes were centrifuged at 0.2 rtf for 15 s. After the 100% ethanol was removed, the cells/clusters were immersed in 100 µL optical clearing agent (Viskol^®^ HISTO-M^™^, Millipore-Sigma, Burlington, MA) for 15 min. These specimen suspensions were transferred to a cover glass with a silicon bank (Grace Bio-Labs, Bend, OR), and then imaged under a laser confocal microscope (FV-1000, Olympus Life Science, Tokyo, Japan). Images of these specimens were acquired using lasers at 405, 473, and 559 nm.

### 2.5 Evaluation of gene expression

Spheroids/clusters and remaining cells in a well were harvested from 30 wells at 1, 2, 7, and 14 days and collected through centrifugation at 1,000 rpm for 5 min for gene expression assays. The pellets were homogenized with a handheld homogenizer pestle (Fisher Scientific, Waltham, MA) in a guanidine isothiocyanate-based extraction buffer (Buffer RLT, RNeasy kit^®^, Qiagen, Valencia, CA) containing 1% β-mercaptoethanol for lysing cells before RNA isolation. Total RNA was extracted following the manufacturer’s instructions for the RNeasy kit^®^. The amount of RNA was determined with a spectrophotometer (NanoDrop ND-1000; Fisher Scientific), and the samples were kept at −80°C until reverse transcription polymerase chain reaction (RT-PCR) was performed. The RNA samples were amplified with RT-PCR using a high-capacity cDNA reverse transcription kit (ThermoFisher) to synthesize cDNA. This cDNA was mixed with TaqMan™ gene expression master mix^®^ (ThermoFisher) and the fluorescent-labeled probe of each desired molecule (TaqMan™ probe, ThermoFisher), followed by measurement of gene expression with real-time PCR (QuantStudio™, ThermoFisher). TaqMan gene expression assays were conducted with aggrecan core protein, *Acan*: Bt03212189_m1; chondroitin sulfate N-acetylgalactosaminyltransferase 1, *Csgalnact1*: Bt03272948_m1; hyaluronan synthase 2, *Has2*: Bt03212694_g1; collagen-type II, *Col2a1*: Bt03251837_mH; collagen-type I, *Col1a1*: Bt03225358_g1; matrix-metalloproteinase-13, *Mmp13*: Bt03214051_m1; proliferating cell nuclear antigen, *Pcna*: Bt03211154_g1; N-cadherin, *Cdh2*; Bt04298958_m1; integrin-V, *ItgaV*: Bt04299013_g1, and glyceraldehyde 3-phosphate dehydrogenase, *Gapdh*: Bt03210919_g1 (Life Technology). Data were analyzed with Expression Suite Software v.1.0.4 (ThermoFisher) to convert ΔCt to relative quantity (RQ).

### 2.6 Definition of parameters for CPM simulating spheroid formation

Spheroid formation by chondrocytes was simulated *in silico* with the CPM using the CompuCell3D simulator [version 4.2.3 (DOI: 10.1093/bioinformatics/bth050), http://www.compucell3d.org] developed by Swat, Glazier, and D'Souza for simulating various cellular events (Glazier and Graner, 1993; Graner and Glazier, 1992; Swat et al., 2012; Vasiev et al., 2010; Shirinifard et al., 2009; Li and Chang, 2014). We used this program to reproduce the formation process of a spheroid with virtual cells and ECM in conjunction with actual cellular events by bACs isolated from specific depth zones in articular cartilage.

The interactions of multiple objects were expressed by total energy, Hamiltonian, H. We defined the H composing contact energy of compartments, H_contact_; and volume constraint of compartments, H_volume_ (Table 1).

$$H=H_{\mathrm{contact}}+H_{\mathrm{volume}}$$

$$H_{contact}=\sum_{\begin{array}{c} \vec{i},\vec{j}\\ neighbors \end{array}}\;J(t(\sigma \vec{i},t\left(\sigma \vec{j}\right)(1\;‐\;d\left(\sigma \vec{i},\sigma \vec{j}\;\right)$$

$$H_{volume}=\sum_{\sigma }\left[\lambda_{\mathrm{vol}}\;\left(\sigma \right)\;{\left(V\left(\sigma \right)\;‐\;V_{\mathrm{target}}\left(\sigma \right)\right)}^{2}\right]$$

**TABLE 1:** Parameters and values used in the cellular potts model.

Parameter	Definition	Cell/ECM type		
		SZ	MZ	DZ
V_Cell_	Target volume of cell (voxel^3^)	30	30	30
V_CellInit_	Initial volume of cell (voxel^3^)	27	27	27
V_A_	Volume of cell where action (division or ECM production) is cued (voxel^3^)	20	20	20
vg	Growth speed of cells (voxel^3^/MCS)	+0.080	+0.060	+0.060
P(M)	Probability that cell undergoes mitosis (%)	1.0	1.0	1.0
V_parent_	Volume of parent cell after mitosis (voxel^3^)	V_Cell_/2	V_Cell_/2	V_Cell_/2
V_daughter_	Volume of daughter cell after mitosis (voxel^3^)	V_Cell_/2	V_Cell_/2	V_Cell_/2
V_Death_	Volume of cell where it is deleted from the simulation (voxel^3^)	>60	>60	>60
P (ECMst)	Probability that cell produces ECMst (%) (calculated from RQ of Col1a1 at day 7)	3.2	0.67	3.0
P (ECMv)	Probability that cell produces ECMv (%) (calculated from RQ of Col2a1 at day 7)	3.2	9.3	8.3
V_ECMInit_	Initial volume of an ECM (voxel^3^)	30	30	30
V_ECM_	Target volume of ECM (voxel^3^)	-	-	-
v_d_	Decay speed of ECM (converted from the RQ of *Mmp13* at day 7)	0.003	0.027	0.097
V_Delete_	Volume of ECM where it is deleted from the simulation (voxel^3^)	<3.0	<3.0	<3.0
λvol	Volume constraint magnitude	10	10	10
Tm	Temperature of domain	37	37	37
*J* (medium-medium)	Contact energy between medium and medium	0	0	0
*J* (cell-cell)	Contact energy between cell and cell (converted from RQ of *Cdh2* at day 7)	5.0	2.5	2.5
*J* (medium-cell)	Contact energy between medium and cell	5.0	5.0	5.0
*J* (medium-ECM)	Contact energy between medium and ECM	20	2.5	2.5
*J* (cell-ECM)	Contact energy between cell and ECM (calculated from RQ of ItgaV at day 7)	10	10	10
*J* (ECM–ECM)	Contact energy between ECM and ECM	10	10	10

ECM, Extracellular matrix; SZ, surface zone; MZ, middle zone; DZ, deep zone.

In the above formulae, H_contact_ represents the contact energy between neighboring compartments (medium-medium, medium–cell, medium–ECM, cell-cell, cell-ECM, and ECM–ECM) over all pairs $\vec{i}$ and $\vec{j}$ in a voxel lattice. In our study, bACs tended to aggregate (evidence of cell-cell contact) without motion *in vitro*. We reproduced this cell aggregation by expressing contact energy, J, cell–cell as being lower than others (medium–cell, medium–ECM, cell-ECM, and ECM–ECM). Thus, we used J (medium-medium) = 0; J (cell-cell) = 2.5 or 5; J (medium–cell), J (cell-ECM), J (ECM–ECM) = 5.0, 10, and 10, respectively defined with the relative value of binding factors.

### 2.7 Initial setup of the simulation and validation

The initial setup of the CPM was designed to recapitulate conditions affecting the spheroids at day 1 based on microscopic images (Figure 2). Each initial cubic size of a cell, structural ECM (ECMst), and volumetric ECM (ECMv) were set as 27 voxel^3^ (3 voxels in width, depth, and height). When 1,000 bACs were seeded to a round-bottom well *in vitro*, the cells settled within a concentric circle-like territory by 4 h at radii, r_init_, which was dependent on cell type (SZ, MZ, or DZ). To recapitulate this cellular dispersion, SZ, MZ, and DZ cells were distributed to cylindrical regions with a diameter of 36, 84, or 84 voxels and height of 5, 3, or 3 voxels, respectively. Note here that the height of the cylinder was defined to fit 1,000 cells within the radii of the clusters observed *in vitro*. ECMst and ECMv were also dispersed within the cylindrical cluster at cell:ECMst:ECMv ratios of 8:1:1 for SZ and 4:4:3 for MZ and DZ. These values were optimized to recapitulate the cell density observed *in vitro*.

In the simulation, the behaviors of individual cells and ECMs were defined based on an iterative algorithm where the state of the cells and ECM were updated every Monte Carlo Step (MCS), with parameters defined based on morphological observation and gene expression of cellular and ECM molecules (Figure 5A). Cells with a volume V_cell_ grow at a rate of 0.080 voxel^3^/MCS. When V_cell_ was larger than the action threshold (V_A_), cells were programmed to undergo division or produce ECM probabilistically. ECM produced by the cells will enter the ECM behavior algorithm *in silico*, which mimics the decay and diffusion present *in vitro* (Figure 5B). ECM decays at v_d_ voxel^3^/MCS every MCS. When the volume of an ECM falls below the deletion threshold (V_D_), the ECM will be deleted from the simulation, and the volume that the ECM had occupied will be replaced by the medium. The growth rate of cells (v_g_), secretion probability of the ECM [P (ECMst) and P (ECMv)], and the decomposition rate of ECM (v_d_) were set using data from investigations of spheroid formation and gene expression *in vitro*. The secretion (synthetic) probability of ECMst [P (ECMst)] was defined as 3.3, 0.67, and 3.0%/MCS for SZ, MZ, and DZ, respectively using the RQ of *Col1a1* at day 7. Similarly, the secretion ratio of ECMv [P (ECMv)], defined using the RQ of *Col2a1* at day 7, was defined as 3.3, 9.3, and 8.3%/MCS for SZ, MZ, and DZ, respectively.

The spheroids *in silico* were incubated for predetermined MCS. The interaction of objects was altered stochastically and proceeded via a Monte Carlo method by repeating an index-copy attempt. Zero MCS *in silico* represented day 1, and 3120 MCS represented day 14 of the *in vitro* cultures. We regarded 1 step as 6 min and 240 steps as 1 day *in vitro*. A simulation (3,120 steps, regarded as 14 days) took about 5–20 h on Windows^®^ personal computers with a 3.3 GHz Intel Core i5^®^ processor.

## 3 Results

### 3.1 Spherical/cell cluster formation of chondrocytes isolated from a specific longitudinal depth zone in cartilage *in vitro*

bACs settled into the U-shaped bottom well in a concentric fashion 4 hours after cell seeding. SZ cells had contact with each other 24 h after seeding, while MZ and DZ cells were still spread at the bottom of the well (Figure 2). By day 4, 100–150 SZ cells were seen in the center region. Meanwhile, aggregations of 5–10 cells were seen at the well bottom with MZ and DZ cells. To avoid disturbance of the suspended cells after seeding, the cells were incubated for 14 days without medium change. The cell viability at days 14 were confirmed to validate cell cultures. Since we used long-working distance objective lens for 3D multicellular mass (spheroids/clusters), accountability of imaging resolution has a limitation. A few MZ cell clusters were seen near the center of the concentrically suspended cells. A DZ spheroid formed at the center region of the well bottom, surrounded by 4–8 smaller satellite spheroids. All spheroids/clusters enlarged over the 14 days of incubation. These cluster formations were not seen on day 1, plausibly attributable to medium convection, which dynamically repositioned the cells during the early stages of culture. After these cells settled to the bottom of the well, actively moving cells were no longer seen.

### 3.2 Accumulation of S-GAG ECM and DNA

**FIGURE 3:**
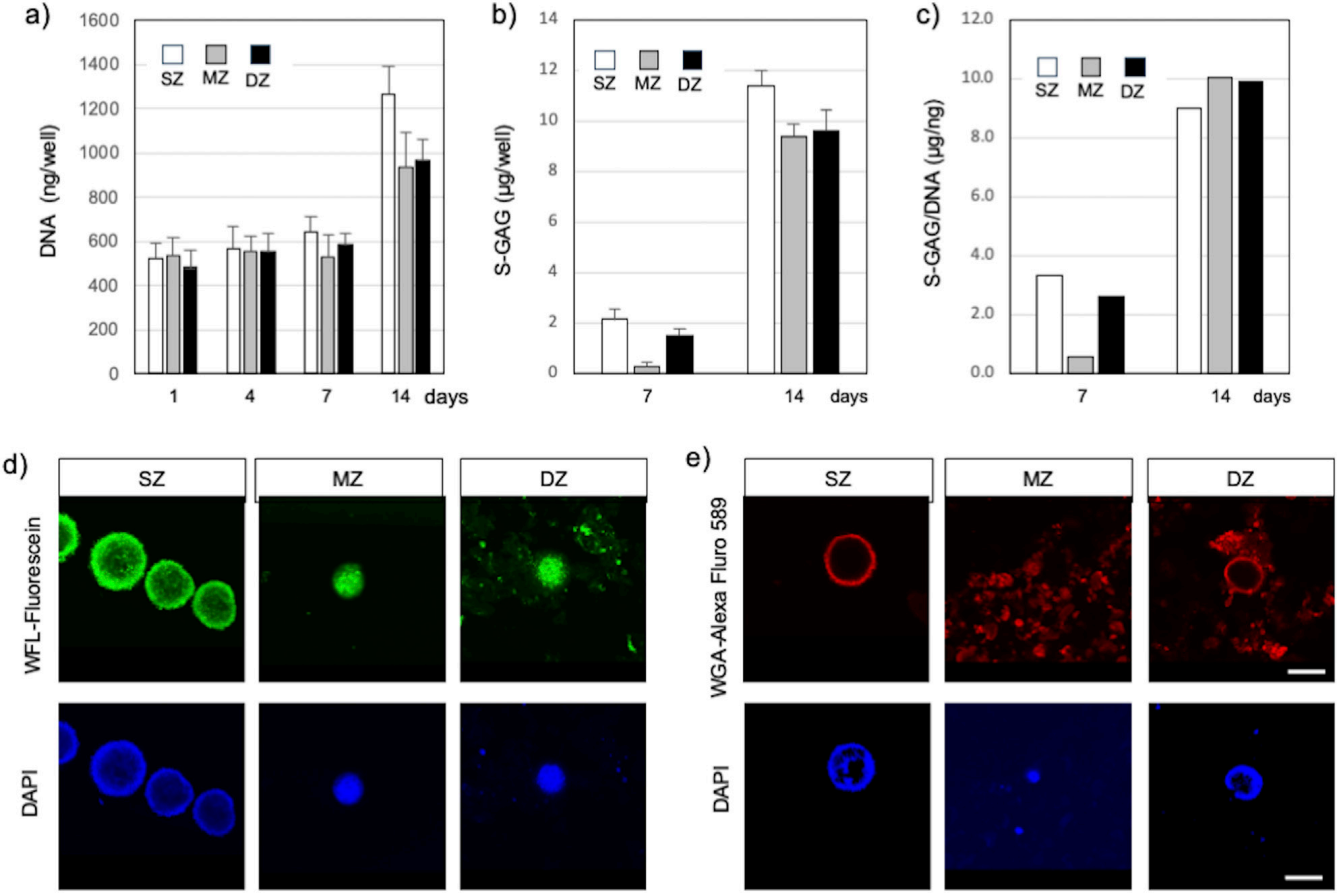
Spheroids and cell clusters formed by chondrocytes isolated from specific zones in bovine articular cartilage at 14 days after cell seeding. **(A)** Amount of DNA, **(B)** Amount of S-GAG, **(C)** Amount of S-GAG/DNA, **(D)** Spheroid/cell clusters stained with Wisteria Floribunda lectin (WFL) – fluorescein at 14 days, **(E)** Spheroid/cell clusters stained with Wheat Germ agglutinin lectin (WGA) – Alexa Fluor 594 at 14 days. Nuclei were counter stained with DAPI. Surface-zone-derived spheroids/cell clusters; SZ, Middle-zone-derived spheroids/cell clusters; MZ, Deep-zone-derived spheroids/clusters; DZ. A bar indicates 100 µm.

S-GAG accumulated in the culture medium was measured to estimate the amount of newly synthesized chondroitin sulfate (Figure 3B). The amount of S-GAG per each well of all cell types remained below the level of detection by the DMMB assay until 4 days. At 7 days, the S-GAG level of all cell types reached detection level. S-GAG in SZ culture was 2.16 µg/well (150 µL medium/well) at 7 days and significantly increased to 11.4 µg/well by 14 days (*P* < 0.01). Similarly, S-GAG in MZ culture was 0.41 µg/well at 7 days and significantly increased to 12.5 µg/well by 14 days (*P* < 0.01). Furthermore, S-GAG in DZ culture was 2.05 µg/well at 7 days and increased to 12.83 µg/well by 14 days (*P* < 0.01). The S-GAG quantified here included those of any molecular weight secreted and accumulated between cells and within culture medium are expected to work as ECMv, because they are non-cell adherent and are capable of retaining abundant water.

The amount of DNA in each well was quantified to estimate cell proliferation and support enlargement of spheroids or cell clusters (Figure 3A). DNA amounts in SZ, MZ, and DZ cells were approximately 543, 531, and 584 ng/well at 7 days, respectively. These values increased significantly by 2.0 (*P* < 0.01), 1.8 (*P* < 0.01), and 1.7 fold (*P* < 0.01) in SZ, MZ, and DZ cultures, respectively, by 14 days. Since each cell within each well had the capability to produce S-GAG, we calculated the S-GAG per DNA (Figure 3C). The difference in S-GAG production rate between cell types decreased over time, and converged to a similar value by day 14.

#### 3.2.1 Localization of CS and HY in chondrocyte spheroids/clusters

SZ cells accumulated CS illuminated with WFL-fluorescein inside and in the outermost layer of spheroids (Figure 3D). A few nuclei illuminated with DAPI were also seen in similar locations. MZ cells formed spheroids/clusters and accumulated CS between the cells and at the periphery of the spheroids, and their nuclei aggregated and compacted. DZ cells also formed spheroids/clusters that accumulated CS in the same manner as the MZ cells. Although we did not intend to stain invisible ECM, WFL-fluorescein stained CS in the ECM precipitation due to centrifugation. Furthermore, SZ cells accumulated HY in the outermost layer of the SZ spheroid but were absent within the spheroid (Figure 3E). MZ cells accumulated HY in and outside of spheroids or clusters where cells were densely co-localized, and in the culture medium. Similarly, DZ cells accumulated HY in the outermost layer of the spheroids/clusters and within the culture medium. However, few nuclei were seen in the medium.

#### 3.2.2 Gene expression representing cellular behavior and extracellular matrix production

**FIGURE 4:**
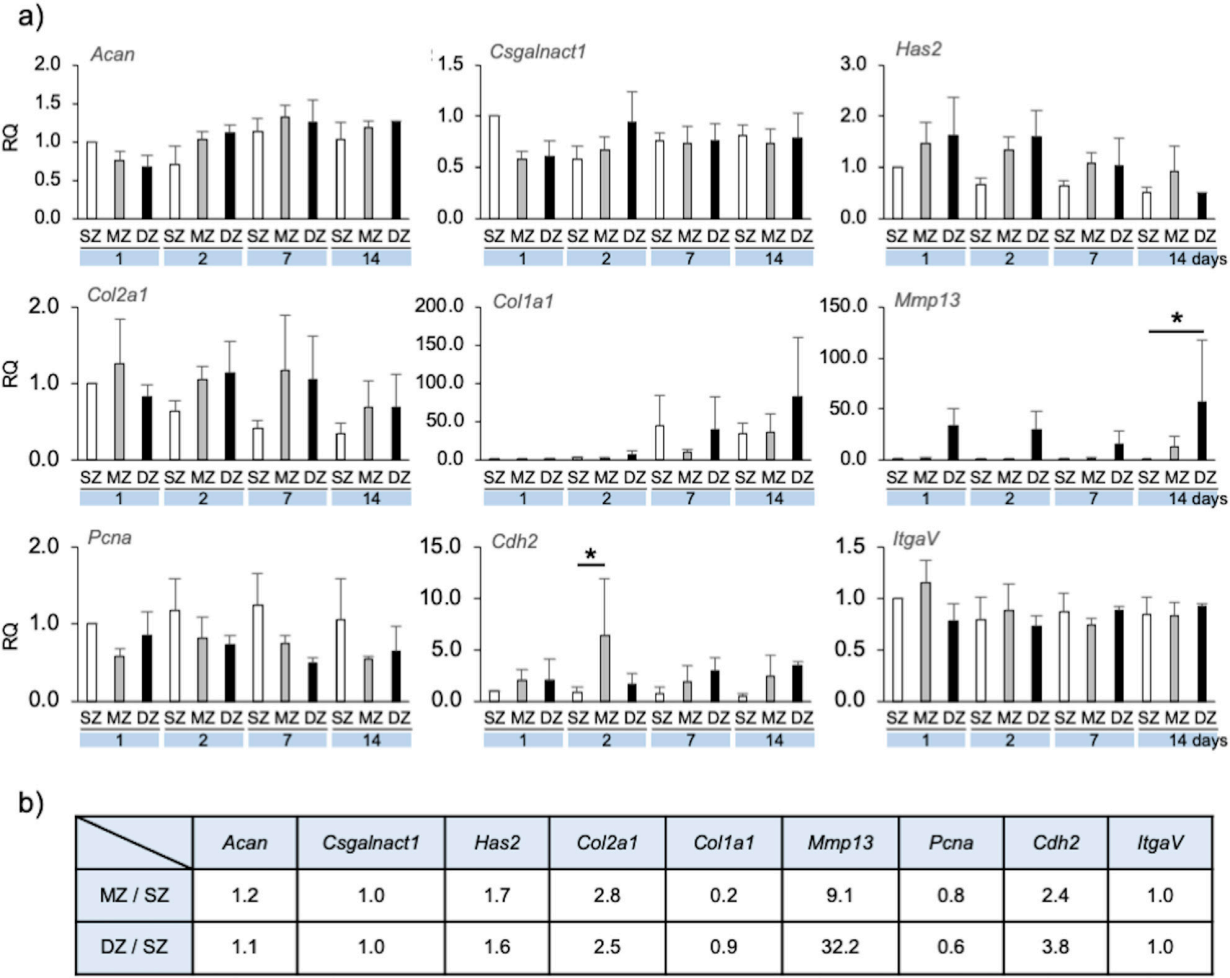
Gene expression by longitudinal depth zone-derived chondrocytes forming spheroids. **(A)** The relative quantity (RQ) of gene expression at 1, 2, 7 and 14 days, compared with that of SZ at day 1. Asterisks indicate significant difference among groups (*p < 0.05, **p < 0.01). A bar indicates SD. **(B)** Ratio of gene expression of MZ and DZ compared with SZ at Day7 for simulation parameter. Acan, Aggrecan core protein; *Csgalnact1*, chondroitin sulfate N-acetylgalactosaminyltransferase 1; *Has2*, hyaluronan synthase 2; *Col2a1*, collagen-type II; *Col1a1*, collagen-type I; *Mmp13*, matrix-metalloproteinase-13; *Pcna*, proliferating cell nuclear antigen; *Cdh2*, N-cadherin; *ItgaV*, integrin-V.

The gene expression of cellular and ECM molecules in chondrocytes during spheroid formation was quantified using RT-qPCR (Figure 4). The relative quantity (RQ) of each gene’s expression at days 2, 7, and 14 was compared with that of SZ at day 1, whose value was defined as 1.0. Distinct zone-specific spheroids were formed by day 7, allowing us to compare RQ among the three zones.

*Acan* and *Csgalnact-1* expression in MZ and DZ spheroid/clusters were both slightly reduced on day 1 compared to SZ and were maintained at approximately 1.0 over 14 days. *Has2* and *Col2a1* expression were approximately 1.5–2 times greater in MZ and DZ compared to SZ spheroids/clusters on days 1, 2, and 7. *Col1a1* expression of SZ and DZ cells at day 7 was strongly upregulated compared to that of MZ. *Mmp-13* expression in SZ and MZ cells was significantly lower than in DZ from days 1–14. *Pcna* expression in SZ cells was approximately 1.5–2 times greater than that in MZ and DZ on days 1, 2, 7, and 14. *Cdh2* expression in SZ cells was lower than in MZ and DZ, and that in MZ at day 2 was significantly upregulated compared to SZ and DZ. No noticeable trend regarding *ItgaV* expression was observed in SZ, MZ, or DZ cells.

#### 3.2.3 Spheroid/cell cluster formation *in silico*

**FIGURE 5:**
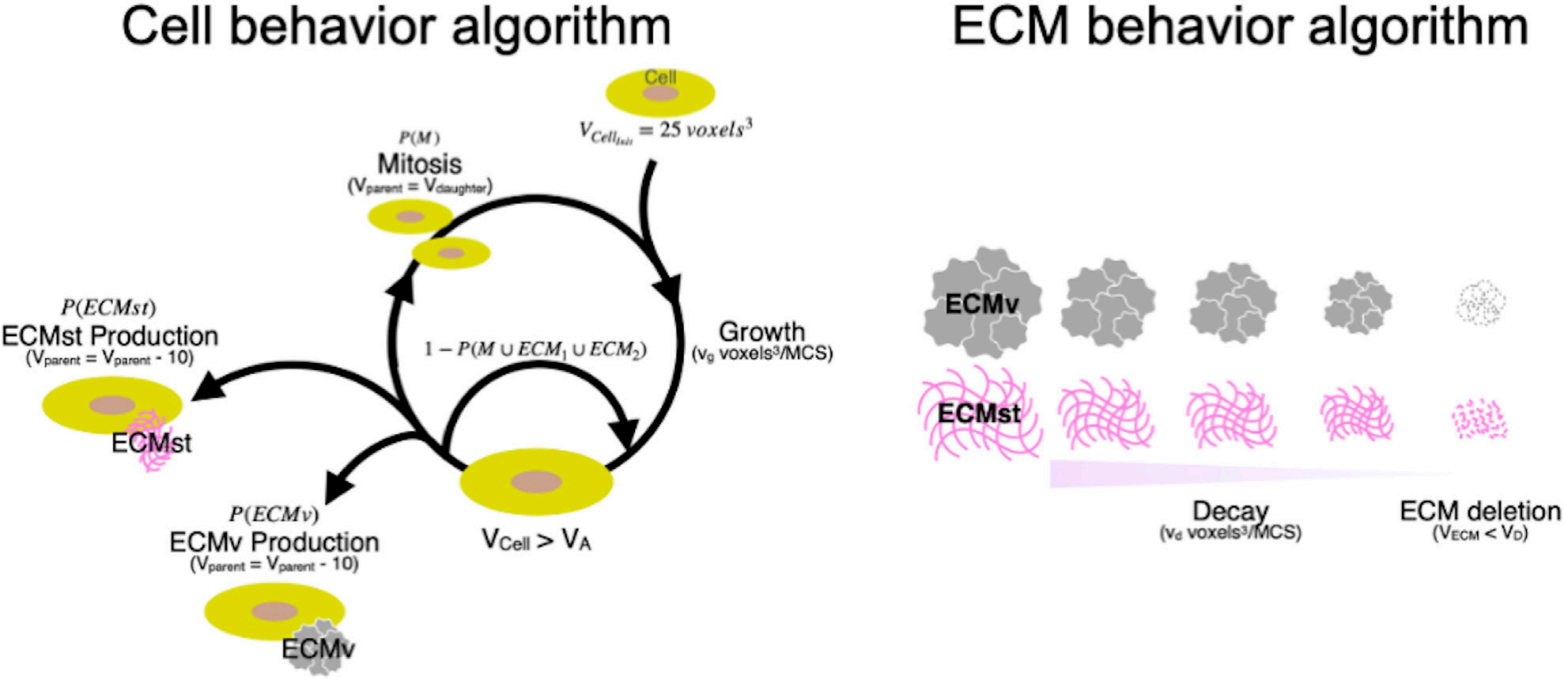
Cyclic cellular and ECM behavior algorithm *in silico*. Initial volume of cell (voxel^3^); V_Cellinit_, Growth speed of cells (voxel^3^/MCS); v_g_, Target volume of cell (voxel^3^); V_cell_, Volume of cell where action division or ECM production is cued (voxel^3^); V_A_, Volume of parent cells after mitosis (voxel^3^); V_parent_, Volume of daughter cell after mitosis (voxel^3^); V_daugther_, Structural ECM; ECMst, Volumetric ECM; ECMv, Probability that cell undergoes mitosis (%); P(M), Probability that cell produces ECMst (%); P(ECMst), Probability that cell produces ECMv (%); P(ECMv), Decay speed of ECM; v_d_, Target volume of ECM; V_ECM_, Volume of ECM where it is deleted from the simulation (voxel^3^); V_Delete_, Monte Carlo steps; MCS.

**FIGURE 6:**
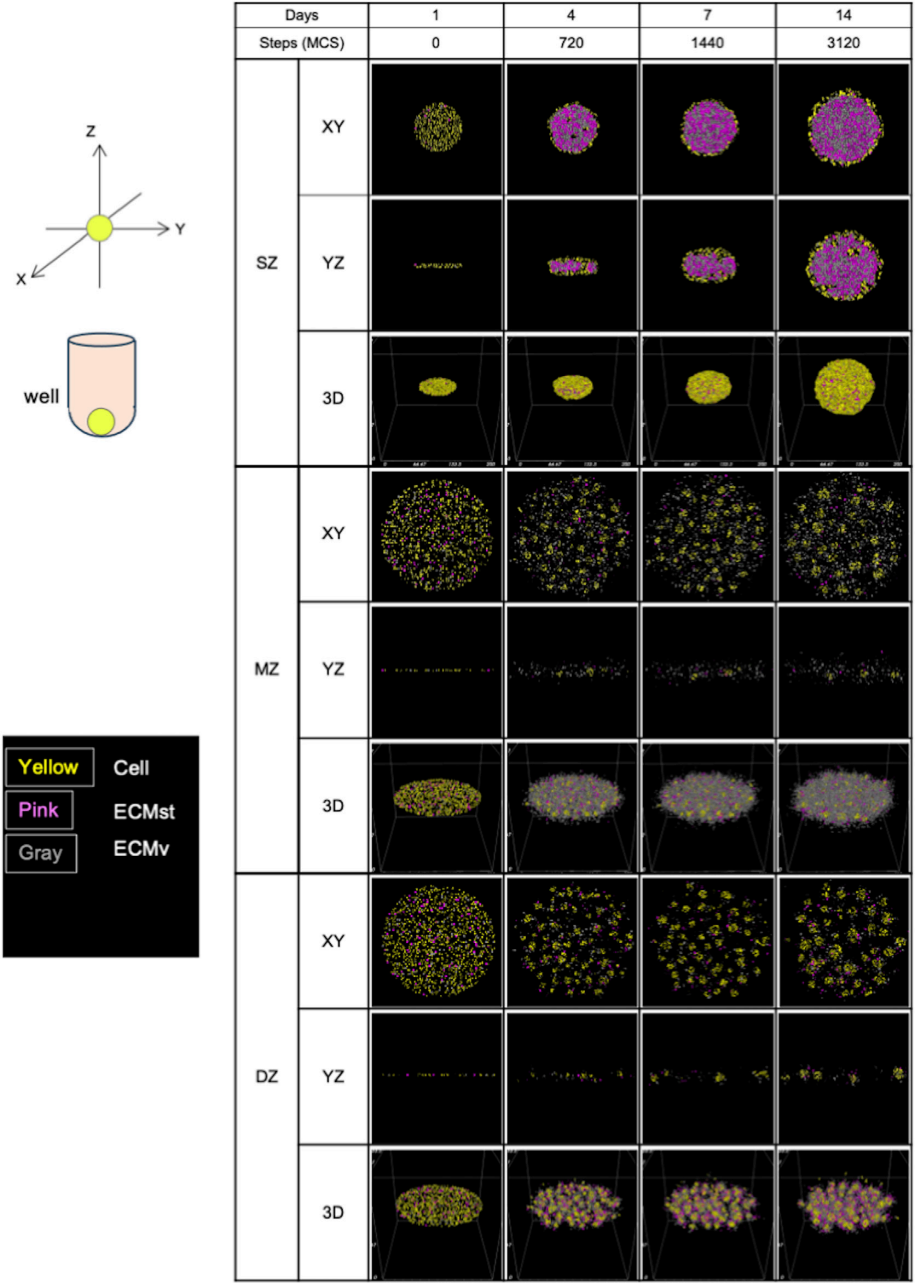
Formation of spheroids and cell clusters *in silico* by virtual chondrocytes isolated from longitudinal depth zone in bovine articular cartilage at days 1, 4, 7, and 14. SZ, surface zone; MZ, middle zone; DZ, deep zone; XY, X-Y axis view; YZ, Y-Z axis view; 3D, Three-dimensional view; MCS, Monte Caro step.

Using the CPM, chondrocyte spheroid formation was simulated with cellular and ECM parameters adopted from the morphology and gene expression of the cells at day 7 (Figures 5, 6; Table 1). We defined two types of ECM: 1) ECMst, representing structural components of ECM, *e.g*., *Col1a1*, and 2) ECMv representing volumetric components, e.g., *Acan*, *Col2a1*, *Csganact1*, and *Has2*. We compared key aspects of the product spheroids at day 14 *in vitro* and *in silico* to semi-quantitatively validate the capability of the *in silico* model to recapitulate spheroid formation *in vitro* (Table 1). To validate the parameters related to cell growth, we checked the diameter ratios of the SZ spheroids at day 1 and day 14 *in vitro* and *in silico*. In case of the parameter in Table 1, the diameter rations of the SZ spheroids at 1 day 14 days were close (1.8 *in vitro* and 2.1 *in silico*). The diameter ratios of the SZ spheroids at days 14 and day 1 *in vitro* and *in silico* were similar (1.8 *in vitro* and 2.1 *in silico*).

## 4 Discussion

Articular cartilage comprises distinct longitudinal depth zones: SZ, MZ, and DZ (Lin and Chang, 2008; Fox, et al., 2009; Fujioka et al., 2013). The articular cartilage is an avascular, aneurogenic, and ECM rich tissue, and the mechanisms of depth zone formation are not known. Therefore, we believe it is a tissue adequately simple to simulate zone specific cellular cluster formation with cell and ECM parameters (Mizuno et al., 2016; Takada and Mizuno, 2018). We demonstrated *in silico* formation of cartilage-depth-zone specific spherical clusters over 14 days then bidirectionally compared it with the formation of spheroids and clusters *in vitro* using cellular and ECM parameters obtained from morphological acquisition and RNA expression at days 7.

### 4.1 Formation of spherical/cell clusters by SZ, MZ, and DZ–derived chondrocytes *in vitro*

The formation of depth zone–specific spheroidal/cell clusters was characterized with cytomorphology and with gene expressions of their formation-related molecules. The distance between neighboring (adjacent) cells in suspension seems to be critical to begin with. It is assumed that the seeded cells are randomly distributed along the low cell binding surface of a round-bottom culture well. Since the cells were seeded at the same density (1,000 cells/150 µL medium), the cells could move in the medium as long as they were in suspension, due to convention in the culture medium. Under suspension, cells were able to reestablish cell-cell contacts orchestrated by cell surface binding molecules such as cadherin and cell-ECM contacts with binding molecules such as integrin (Woods et al., 2007). We previously used an anti-cadherin antibody to identify the location of cadherin and an anti-integrin antibody to identify the location of integrin, demonstrating cell-ECM contact (Mizuno, et al., 2016). Meanwhile, the cells were surrounded with newly synthesized non-cell-binding ECM, e.g., HY and CS, defined as ECM volumetric factors (ECMv), which prevents further direct cell-cell binding; and cell-binding ECM, e.g., collagens defined as ECM structural factors (ECMst), which recruits adjacent cells. Though ECMs were invisible under regular inverted microscopy, the presence of these ECMs was supported by our observation of the slime-like ECM that pulled cells during aspiration of the culture medium with a fine pipette tip. In particular, MZ- and DZ-derived cells demonstrated a strong capacity to produce abundant ECMs stained with WGA-Alexa Fluor 594 and greater expressions of *Col1a1* and *Has2* in SZ and DZ cells. Our observations indicated that these two cell types formed larger spheroids than MZ cells. This result closely matches the results from past literature where tumor cells cultured in medium with high collagen concentration formed larger and denser clusters due to the hinderance of cell migration (Gonçalves and Garcia-Aznar, 2021). Thus, it is evident that the synthetic capability of ECMs and their localization greatly affect the volume and number of SZ, MZ, and DZ spheroids and cell clusters. These translations were also supported by cytomorphological observation under confocal laser scanning microscopy (CLSM), with staining conducted using WFL-Fluoresceine and WGA-Alexa Fluor 594, each binding to N-acetylgalactosamine (indicating CS, Mueller et al., 2014) and N-acetylglucosamine (indicating HY, Hsu et al., 2007). SZ cells formed a spheroid earlier than other cell types (day 4) and accumulated CS at both the core and surface layer of the spheroid, whereas HY accumulated only in the outermost layer of the spheroid, and formed a spherical compartment with proliferating cells (Figures 3D, E). On the other hand, MZ and DZ cells accumulated CS between cells and in the medium, though small spheroids and clusters were formed. S-GAG/DNA indicated that each cell produced CS at similar levels. Thus, it can be safely concluded that MZ and DZ cells secreted a greater amount of CS and HY into the medium, which is consistent with the observations made under CLSM using WFL-Fluorescein and WGA-Alexa Fluor 594 lectins.

### 4.2 Spheroidal/cell cluster formation *in silico*

We simulated the formation of chondrocyte spherical/cell cluster using the CPM with cellular and ECM factors converted from gene expression by specific longitudinal zone–derived chondrocytes at day 7 *in vitro* (Figure 4B; Table 1).

#### 4.2.1 Initial cell orientation

Similar to the seeding procedure *in vitro*, 1,000 cells were deployed *in silico* for all cell types, but oriented to mimic the situation of *in vitro* cells at day 1 (Figure 2). As was observed *in vitro*, MZ and DZ cells formed a flatter surface, while SZ cells were positioned more closely to each other, resulting in a thicker, cylindrical arrangement at 0 MCS. Although a side-view image of the initial cell orientation *in vitro* cannot be obtained, this 3D distribution is a plausible arrangement in the U-bottom well when cells are seeded.

### 4.3 Progression of spherical cluster formation

Initially, all *in silico* cell cultures in this study were homogeneously dispersed on the virtual culture substrate, appearing as thin multi-layered cell suspensions (Figure 6). After 720 MCS, the cells started to aggregate into 3D structures in a depth zone–specific cell orientation. SZ cells were connected mainly at the surface of the formed spheroid, which can be explained by the high levels of cell-cell binding factor, J (SZ cell–SZ cell), and higher ECMst productivity compared to the other 2 cell types (Table 1). With substantial ECMst, the spheroid had a high loading capacity for ECMv due to the direct binding between ECMst and ECMv (Douglas et al., 2007), leading to a greater accumulation of ECM. Once SZ cells contact each other, external force from the environment is required to dissociate them. When ECMv began accumulating inside of the developing spheroid, that initiated an outward force, which contributed to the stretch of the tightly connected cells, triggering the expansion of the spheroid. Cells that were not located at the surface of the spheroid remained unaffected by the increasing ECM. Based on the cross-section histology of the SZ spheroid previously reported (Mizuno et al., 2016), we believe that the cellular and ECM factors chosen reproduced SZ formation *in silico*. It should be noted that these observations differ from those of spheroids formed from chondrocytes derived from all depth-zones, where both chondrocytes and ECM accumulation were seen at the core (Omelyanenko et al., 2020). This suggests that the cells from the three depth-zones and the ECM they produce interact and complement each other to form the native morphology.

After 720 MCS, in contrast to SZ cells, MZ cells (which have higher cell-cell binding affinity and ECM productivity) displayed multiple cell “clusters,” with low ECM accumulation at the spheroid cores. One potential reason for this lower ECM accumulation in comparison to SZ cells is the higher MMP-13 expression, which is a collagenase known to cause distint ECM degradation (Solomonov et al., 2016). The scarcity of ECMst (shown in pink in Figure 5) between the cell clusters also contributed to preventing the MZ spheroids from accumulating ECM. Lack of ECMst accumulation, caused by diffusion out to the fluid medium, reduced the spheroids’ capability to hold ECMv and the cells together. This in turn may have contributed to the formation of multiple cell clusters (Figure 2).

DZ spheroids showed a mixture of the characteristics of SZ and MZ cells. Though MZ and DZ cells had the same cell-cell contact factor (Table 1), DZ cells formed larger cell clusters (Figure 2). This is presumably due to the faster production of ECMst, which held the cells and ECMs together, leading to the accumulation of ECM at the core of the DZ spheroids. Compared to SZ spheroids, DZ spheroids were smaller despite their higher ECMv production rate. This difference may also be a product of the higher expression of MMP-13, which contributed to the faster decay of ECM in DZ spheroids. The higher cell-cell contact factor between DZ cells also made the spheroids less flexible, allowing less ECM to accumulate at the core. The images of spheroid formulation also imply that after the cells formed clusters, the clusters grew in size not by accumulating ECM, but instead by merging with neighboring clusters. Thus, the cores of the DZ spheroids were filled with more cells while the cores of SZ spheroids were filled with abundant ECM (Mizuno et al., 2016). Although *in vitro* SZ and DZ spheroids appeared somewhat similar under bright field microscopy, we acknowledge that their content may differ greatly. Nevertheless, the ability to visualize the section of the spheroids in a non-destructive manner is a significant advantage of *in silico* models, making such models useful for various applications.

### 4.4 Morphology of spheroids/clusters at 3120 MCS

The *in vitro* morphology and *in silico* spheroids were very similar, despite the fact that only a few out of the 21 implemented simulation parameters differed among the three cell types (Table 1). The 3D cellular orientation of the spheroids *in silico* was evaluated at 3120 MCS, which corresponds to 14 days *in vitro*.

*In silico*, SZ cells formed a spheroid that had a core-shell orientation, with cells densely aligned along the circumference of the spheroid and the core densely packed with ECM. The morphology of the spheroids was initially a disk-shaped aggregation but evolved into a spherical shape. This is thought to be due to the balance between the enlarging force emanating from the center of the spheroid outwards (due to the accumulation of ECM) and the tensegrity produced by the connectivity of cell-cell contact. The validity of these simulations is supported by the gene expression of *Cdh2* indicating cell-cell contact and *Acan* indicating ECMv. This regulation of spheroid growth by cadherin-dependent cell-cell interaction and outward expansive force was also demonstrated in a glioblastoma model (Ang et al., 2024).

In contrast, MZ cells showed multiple cell aggregations spread across the XY axis with ECMv between the aggregates. It is important to note that the ECM was mostly ECMv and not ECMst, due to the lower productivity of ECMst compared to that of SZ and DZ. Such ECMv, composed of aggrecan and type 2 collagen, is suspected to be the slime-like substance observed at medium exchange. None of the cell aggregates showed significant ECM accumulation at their core. The lower contact energy *J* (MZ cell–MZ cell) in comparison to *J* (SZ cell-SZ cell) may have produced the tight MZ cell aggregates, which left no room for ECM to accumulate.

*In silico*, DZ cells showed minor yet noticeably greater accumulation of ECM within the spheroids compared to MZ cells, although *J* (DZ cell-DZ cell) was the same as that of MZ cells. The ECMv productivity of DZ cells was slightly lower than that of MZ cells, whereas the productivity of ECMst was 3 times higher than that of MZ cells. Collectively, the type of ECM (ECMst and ECMv) and its accumulation rate strongly influence morphology.

Comparing SZ spheroids to DZ spheroids, only SZ spheroids exhibited a remarkable increase in size, even though the ECMv productivity of DZ cells was 3-fold higher. We speculate that this difference had two causes: higher decay constant (v_d_) of ECM and higher cell-cell contact energy *J* (DZ cell-DZ cell) compared to *J* (SZ cell-SZ cell). Since the decay constant of ECM, converted from the gene expression of MMP-13, was 32-fold higher with DZ cells compared to SZ cells, the newly synthesized ECM was degraded at a higher rate. In addition, the high *J* (DZ cell-DZ cell) elicited greater tensegrity to conserve the spheroid volume. Consequently, the outward force exerted by ECM accumulation and the inward force due to tensegrity balanced at a smaller volume, rendering smaller DZ spheroids compared to SZ spheroids. As such, to precisely shape the cell microenvironment, it is crucial to accurately define the cell-cell contact factor, cell-ECM contact factor, ECM productivity, and ECM decay constant, as these elements synergize in a complex manner.

### 4.5 Advantages and limitations of *in silico* modeling

Through a series of *in vitro* and *in silico* experiments, multiple limitations and advantages of the *in silico* model were demonstrated. The phenotype of *in silico* cells was established at the beginning of the simulation determined by the gene expression on day 7 and remained constant throughout. Therefore, future improvements must be made so that the cells can change their phenotype and behavior depending on the dynamic environment. However, it is still beneficial that this *in silico* model required a maximum of only a day to monitor the spheroid morphogenesis over 14 days using currently available hardware (Simian and Bissell, 2019; Dahl-Jensen and Grapin-Botton, 2017). Thus, the time cost of performing spheroid formation studies can be significantly lower compared to *in vitro* models. In addition, changing the values of the already installed parameters, *e.g*., *J* (cell-cell), P (ECMv) can be performed quickly without *in vitro* data, which makes such an *in silico* model a powerful, inexpensive tool in simulating the behavior of spheroids formed by cells at arbitrary conditions. In the current study, the *in silico* model was superior to *in vitro* models, in the sense that the 3D conformation of the spheroids could be viewed and that slice images could be obtained without additional processing of the spheroid. Such advantages will greatly assist in revealing the characteristics of spheroids that cannot be visualized using conventional *in vitro* approaches. Extended applications can be explored for developing new therapeutic modalities using human derived cells *in vitro* and minimizing animal models under the Federal Drug Administration Modernization Act 2.0 (Nuwer, 2022; Stresser et al., 2023). Spherical human chondrocyte clusters shed light on modeling diseases or therapeutic approaches. Incubating with inflammatory factor, e.g., Interleukin 6 (IL-6), DZ cell clusters will likely degrade compared to without IL-6. With the presence of IL-6, collagen type-I in DZ cluster will degrade due to synergistic stimulation with upregulated *Mmp-13*. To simulate this *in vitro* cellular behavior and ECM degradation with *in silico* model, the decay ratio of ECMst can be manipulated to speed up and resulted in reproducing the pathology of osteoarthritis. Spheroids derived from MZ and DZ *in vitro* had notably wide distribution in terms of both number and size. To pursue reproducible numbers and sizes, we carefully conducted cell isolation and consistently monitored them. Additional consideration should be directed toward strictly unifying the duration of enzymatic digestion and the inter-lot variability of collagenase, which includes two other enzymes. These enzymatic variations may cause inconsistent density of pericellular ECM and cell membrane adhesion molecules. After the cells were seeded, we maintained them in a U-bottom low-cell-adhesive-surface well for 14 days without medium change, which minimized disturbance. However, medium convection was not taken into consideration, which could affect the fluid dynamics of the culture medium. Once the cells settle down on the U-bottom well, medium convection is the only potential element that may influence cellular and matrix motion. Thus, the effects of fluid dynamics in culture medium may need to be involved for a further precise *in silico* simulation.

## 5 Conclusion

We consistently reproduced the formation of multicellular spheroids from chondrocytes isolated from specific longitudinal depth zones in articular cartilage *in vitro* and simulated formation *in silico* by optimizing the cellular and ECM affinity factors. We successfully recapitulated spheroid formation by modifying the original version of the CPM with additional cellular and ECM factors. For reproducing comparable *in vitro*–*in silico* morphogenic models, determining not only the cellular characteristics of the chondrocytes of each zone but also their ECM appears essential. Though we still must assess spheroid formation with combined multiple cell types (SZ, MZ, and DZ cells) to elucidate longitudinal zone morphogenesis, our *in silico* strategy using a spherical model has the potential to reproduce point-symmetrically organized morphogenesis within a spheroid. Further implementation of parameters relating to physicochemical nature, synthetic productivity, cell-ECM interaction, etc., would yield a more physiologically relevant tissue model that could contribute to elucidating the decisive factors causing regenerative or degenerative changes with/without biologically potent molecules *in vivo*. Those biological events can be virtually reproduced within minutes on a computer *in silico*.

## Data Availability

The original contributions presented in the study are included in the article/supplementary material, further inquiries can be directed to the corresponding author.

## References

[B1] Abraham D. M. Herman C. Witek L. Cronstein B. R. Flores R. L. Coelho P. G. (2022). Self-assembling human skeletal organoids for disease modeling and drug testing. J. Biomed. Mater Res. B Appl. Biomater. 110, 871–884. 10.1002/jbm.b.34968 34837719 PMC8854332

[B2] Ang I. Yousafzai M. S. Yadav V. Mohler K. Rinehart J. Bouklas N. (2024). Elastocapillary effects determine early matrix deformation by glioblastoma cell spheroids. Apl. Bioeng. 8, 026109. 10.1063/5.0191765 38706957 PMC11069407

[B3] Dahl-Jensen S. Grapin-Botton A. (2017). The physics of organoids: a biophysical approach to understanding organogenesis. Development 144, 946–951. 10.1242/dev.143693 28292839

[B4] Dönges L. Damle A. Mainardi A. Bock T. Shönenberger M. Matrin I. (2024). Engineered human osteoarthritic cartilage organoids. Biomaterials 308, 122549. 10.1016/j.biomaterials.2024.122549 38554643

[B5] Douglas T. Heinemann S. Mietrach C. Hempel U. Bierbaum S. Scharnweber D. (2007). Interactions of collagen types I and II with chondroitin sulfates A-C and their effect on osteoblast adhesion. Biomacromolecules 8, 1085–1092. 10.1021/bm0609644 17378603

[B6] Fox A. J. S. Bedi A. Rodeo S. A. (2009). The basic science of articular cartilage: structure, composition, and function. Sports Health 1, 461–468. 10.1177/1941738109350438 23015907 PMC3445147

[B7] Fujioka R. Aoyama T. Takakuwa T. (2013). The layered structure of the articular surface. Osteoarthr. Cartil. 21, 1092–1098. 10.1016/j.joca.2013.04.021 23680879

[B8] Glazier J. A. Graner F. (1993) Simulation of the differential adhesion driven rearrangement of biological cells biological cells. Phys. Rev. E 47:2128–2154. 10.1103/PhysRevE.47.2128 9960234

[B9] Gonçalves I. G. Garcia-Aznar J. M. (2021). Extracellular matrix density regulates the formation of tumour spheroids through cell migration. PLoS Comput. Biol. 17, e1008764. 10.1371/journal.pcbi.1008764 33635856 PMC7968691

[B10] Graner F. Glazier J. A. (1992). Simulation of biological cell sorting using a two-dimensional extended Potts model. Phys. Rev. Lett. 69, 2013–2016. 10.1103/PhysRevLett.69.2013 10046374

[B11] Harjanto D. Zaman M. H. (2013). Modeling extracellular matrix reorganization in 3D environments. PLoS ONE 8, e52509. 10.1371/journal.pone.0052509 23341900 PMC3544844

[B12] Hsu T. L. Hanson S. R. Kishikawa K. Wang S. K. Sawa M. Wong C. H. (2007). Alkynyl sugar analogs for the labeling and visualization of glycoconjugates in cells. Proc. Natl. Acad. Sci. U.S.A. 104, 2614–2619. 10.1073/pnas.0611307104 17296930 PMC1815231

[B13] Lamandé S. R. Ng E. S. Cameron T. L. Kun L. H. W. Sampurno L. Rowley L. (2023). Modeling human skeletal development using human pluripotent stem cells. Proc. Nat. Acad. Sci. U.S.A. 120, e2211510120. 10.1073/pnas.2211510120 PMC10175848 37126720

[B14] Li J. F. Lowengrub J. (2014). The effects of cell compressibility, motility and contact inhibition on the growth of tumor cell clusters using the Cellular Potts Model. J. Theor. Biol. 343, 79–91. 10.1016/j.jtbi.2013.10.008 24211749 PMC3946864

[B15] Lin R. Z. Chang H. Y. (2008). Recent advances in three-dimensional multicellular spheroid culture for biomedical research. Biotechnol. J. 3, 1172–1184. 10.1002/biot.200700228 18566957

[B16] McCauley H. A. Wells J. M. (2017). Pluripotent stem cell-derived organoids: using principles of developmental biology to grow human tissues in a dish. Development 144, 958–962. 10.1242/dev.140731 28292841 PMC5358106

[B17] Mizuno S. Takada E. Fukai N. (2016). Spheroidal organoids reproduce characteristics of longitudinal depth zones in bovine articular cartilage. Cells Tissues Organs 202, 382–392. 10.1159/000447532 27654347

[B18] Mueller A. Davis A. Carlson S. S. Robinson F. R. (2014). N-acetylgalactosamine positive perineuronal nets in the saccade-related-part of the cerebellar fastigial nucleus do not maintain saccade gain. PLOS One 9, e86154. 10.1371/journal.pone.0086154 24603437 PMC3945643

[B19] Nuwer R. (2022). US agency seeks to phase out animal testing. Nature. 10.1038/d41586-022-03569-9 36333602

[B20] Omelyanenko N. P. Karalkin P. A. Bulanova E. A. Koudan E. V. Parfenov V. A. Rodionov S. A. (2020). Extracellular matrix determines biomechanical properties of chondrospheres during their maturation *in vitro* . Cartilage 11, 521–531. 10.1177/1947603518798890 30221989 PMC7488948

[B21] Scianna M. Preziosi L. (2013). in Cellular Potts models (United States: Chapman and Hall/CRC).

[B22] Shirinifard A. Gens J. S. Zaitlen B. L. Poplawski N. J. Swat M. Glazier J. A. (2009). 3D multi-cell simulation of tumor growth and angiogenesis. PLoS One 4, e7190. 10.1371/journal.pone.0007190 19834621 PMC2760204

[B23] Simian M. Bissell M. J. (2019). Organoids: a historical perspective of thinking in three dimensions. J. Cell. Biol. 216, 31–40. 10.1083/jcb.201610056 PMC5223613 28031422

[B24] Solomonov I. Zehorai E. Talmi-Frank D. Wolf S. G. Shainskaya A. Zhuravlev A. (2016). Distinct biological events generated by ECM proteolysis by two homologous collagenases. Proc. Natl. Acad. Sci. U. S. A. 113, 10884–10889. 10.1073/pnas.1519676113 27630193 PMC5047162

[B25] Stresser D. M. Kopec A. K. Hewitt P. Hardwick R. N. Van Vleet T. R. Mahalingaiah P. K. S. (2023). Toward *in vitro* models for reducing or replacing the use of animals in drug testing. Nat. Biomed. Eng. 10.1038/s41551-023-01154-7 38151640

[B26] Swat M. H. Thomas G. L. Belmonte J. M. Shirinfard A. Hmeljack D. Glazier J. A. (2012). Multi-scale modeling of tissues using CompuCell3D. Methods Cell. Biol. 110, 325–366. 10.1016/B978-0-12-388403-9.00013-8 22482955 PMC3612985

[B27] Takada E. Mizuno S. (2018). Reproduction of characteristics of extracellular matrices in specific longitudinal depth zone cartilage within spherical organoids in response to changes in osmotic pressure. Int. J. Mol. Sci. 19, 1507. 10.3390/ijms19051507 29783650 PMC5983583

[B28] Vasiev B. Balter A. Chaplain M. Glazier J. A. Weijer C. J. (2010). Modeling gastrulation in the chick embryo: formation of the primitive streak. PLoS One 5, e10571. 10.1371/journal.pone.0010571 20485500 PMC2868022

[B29] Voss-Bohme A. (2012). Multi-scale modeling in morphogenesis: a critical analysis of the Cellular Potts Model. PLOS ONE 7 (9), e42852. 10.1371/journal.pone.0042852 22984409 PMC3439478

[B30] Woods A. Wang G. Beier F. (2007). Regulation of chondrocyte differentiation by the actin cytoskeleton and adhesive interactions. J. Cell. Physiol. 213, 1–8. 10.1002/jcp.21110 17492773

